# Orthorexia Nervosa in Adolescents and Young Adults: A Literature Review

**DOI:** 10.3390/children9030365

**Published:** 2022-03-04

**Authors:** Maria Gkiouleka, Christina Stavraki, Theodoros N. Sergentanis, Tonia Vassilakou

**Affiliations:** 1 Department of Public Health Policy, School of Public Health, University of West Attica, Athens University Campus, 11521 Athens, Greece; mariagkiouleka@outlook.com (M.G.); tinastavraki@gmail.com (C.S.); tsergentanis@uniwa.gr (T.N.S.); 2 Health Centre of Nea Kallikrateia, 63080 Nea Kallikrateia, Greece; 3 2nd Primary School of Nafpaktos, 30300 Nafpaktos, Greece

**Keywords:** orthorexia, orthorexia nervosa, adolescent or young adults (AYAs), eating disorders, adolescents, young adults

## Abstract

Adolescents are a nutritionally vulnerable population; eating disorders are more common among adolescents and young adults. Orthorexia nervosa (ON) is a non-formally recognized condition characterized by an obsessive preoccupation with eating healthy and “pure” foods; the quality and not the quantity of food is pivotal in ON. ON is a complex entity which can be associated with severe diet restrictions, a negative impact on social relationships, and with physical and mental health conditions, including obsessive–compulsive disorder. In light of this, a literature review regarding the background, diagnosis, features, risk factors, interplay with the social media, and management of ON is presented in this article, with a focus on adolescents and young adults.

## 1. Introduction

Adolescence is a period of important biological, hormonal, psychological, mental, and social changes [1]. Moreover, adolescence and early youth are periods of elevated energy and nutrient requirements, necessary in order to meet the increased demands of development and promote optimal physical, cognitive, and psychological health [2]. Furthermore, adolescence is a challenging period for a person’s nutrition, as adolescents are vulnerable to negative body image, weight pressures, and disordered eating behaviors [3], which may be associated with different forms of malnutrition, such as undernutrition, obesity, and deficiencies of certain nutrients [4,5]. In addition, adolescence is a vital time for establishing eating habits that will likely last during a person’s life [6].

The adoption of a balanced eating pattern plays a key role in the development of a healthy lifestyle, as it contributes to decreases in the prevalence of obesity, and offers protection against the development of diabetes mellitus and cardiovascular diseases among numerous other benefits [7]. However, for some people the obsession for healthy or “clean” foods exclusively is likely associated with serious problems both in their health and their social lives [8]. During the last two decades, there has been an increased interest with reference to people becoming obsessed with a healthy diet; a condition known under the term orthorexia nervosa (ON) [8]. Although adopting healthy eating habits, considered as ‘healthy orthorexia’, could be a protective factor to one’s health, imposing strict limitations regarding the purity of foods may be associated with physical and/or mental health conditions [9]. For people with ON the quality of food, not the quantity, as well as the advantages of “clean” eating, is of fundamental significance [8,10].

Orthorexic people usually get anxious when their food comes from sources of ambiguous quality, they could become fixated on the preparation of their meals, following inflexible rules made by themselves, and they tend to express feelings of superiority towards other people who do not share the same eating habits [10]. As ON is not recognized officially as a distinct ED, several gaps exist regarding the etiology, the risk factors and the manifestations of this condition, its relationship with other EDs or restrictive eating patterns, energy and nutrient deficiencies and level of adherence to dietary recommendations, as well as its long-term associations with physical and mental health [8]. Socioeconomic factors and the social media pressure may be associated with an unrealistic body image, desire for thinness, and various unhealthy weight-control methods [7], whereas online information concerning the adoption of healthy food habits is abundant [8,11]. The present article aims to review the existing literature on ON, in the context of the most common EDs faced by children, adolescents and young adults, to emphasize ON predictors and the existing gaps in terms of further research and nutritional interventions regarding early prevention and treatment of this condition.

## 2. Materials and Methods

An electronic search of international literature was carried out, using the MEDLINE and Science Direct databases, accessed through PubMed and Elsevier, respectively, in order to track relevant original studies, reviews, systematic reviews, and meta-analyses published between January 2004 and January 2022. Search terms and combinations used included “orthorexia”, “orthorexia nervosa”, “orthorexia and eating disorders”, “orthorexia and adolescents or young adults”, and “orthorexia nervosa and adolescents or young adults”. Included studies provided data regarding ON among children, adolescents and young adults, as well as the interrelation of ON with other EDs and associations with social media use. All abstracts and full texts were reviewed for relevance. Articles not written in English or Greek were excluded.

## 3. Results

### 3.1. ON: Definition and Main Features

ON is characterized by an unhealthy obsession for healthy eating that emphasizes following extreme rules on a “clean and pure” nutrition and on unreasonable specifications for food selection and/or preparation [12]. People with ON present obsessions and compulsive behavior over the purity of their foods and compliance to the strict dietary rules they impose on themselves. Their adherence to the origin of the food ingredients, the methods used in the cultivation of vegetables, fruits, cereals, and grains, and the preparation of processed foods, as well as to the labels of food packages, are some of the most significant characteristics of ON as an unhealthy eating pattern [13]. Orthorexics try to avoid food treated with chemicals, artificial sweeteners and proteins, or significant amounts of harmful ingredients, such as saturated fats, salt, or sugar, at any cost and they focus excessively on techniques and materials that are involved in healthy food preparation [10,14]. In addition, ensuring and maintaining food purity becomes their goal, as opposed to confining the quantity of the consumed foods and the extreme calorie restriction that are associated with anorexia AN or BN [15]. In severe cases, dietary violations might trigger an attitude for self-punishment, which is manifested by supposedly cleansing fasts or even stricter diets in order to achieve purification [12].

ON might be evidenced by severe dietary restrictions with emphasis on reverent, almost ascetic adherence to a specific diet, so that the patient feels clean and pure and violation of the rules set by the individuals themselves, are associated with a strong feeling of fear of disease, accompanied by anxiety, shame, and a sense of personal impurity. Dietary restrictions might escalate over time, with the exclusion of whole food groups, frequent periods of fasting and deprivation, which are thought to purify or “detoxify” the orthorexics. This gradual escalation could be associated with weight loss, which, however, is overshadowed by the individual’s attachment to a healthy diet [9,16].

Although ON might start as an innocent attempt to eat more healthily, in which attention to food selection is not pathological itself, it might turn into a highly absorbing obsession that penetrates almost every aspect of orthorexic individuals’ lives [17]. People with ON tend to spend a considerable amount of money and energy in order to plan meals in advance, buy all their foods after cautious reading of the labels about energy and nutrients’ content, meticulously select grocery stores and, eventually, to prepare home-cooked meals in reverent devotion [15]. Orthorexic subjects also pay great attention in warning messages provided by the media about possible threats associated with the consumption of certain foods or the intake of various food ingredients and food additives present in processed foods and in advertisements of low-fat, high-protein, organic, gluten-free, sugar-free, lactose-free, or salt-free foods [14], while a significant amount of time is also spent on food-related thoughts [12]. This dietary extremism, due to omission of entire food groups, might be associated with severe nutritional deficits and complications similar to those with AN [14,18].

On a social level, orthorexic behavior could disrupt orthorexic persons’ relations with others, social interests, and activities, as well as their involvement and participation with friends and family in social events [12,13], in school, work, and social lives [19]. They might distance themselves from others, feeling that nobody really understands their constant need to gain control over “clean eating”, and sometimes they could become incapable of having normal social and professional interactions with others [19]. In addition, avoidance of accepting invitations to social events that include or may include gathering around a table could be gradually associated with social exclusion [20].

### 3.2. Origin of the Term “Orthorexia Nervosa”

The scientific term “orthorexia nervosa” is relatively recent. It was coined in 1997 by Steven Bratman, a physician specialized in alternative medicine. It first appeared in his article “Health Food Junkie” that was published in the non- scientific “Yoga Journal” magazine, in order to describe the pathological obsession of certain people with dietary perfection. Etymologically the term comes from the merger of the Greek words “ortho”, which means correct-proper, and “orexis” that means appetite [12]. The term “orthorexia nervosa” was introduced in terminology in an Italian study which established orthorexia as a condition that needed further scientific evaluation [13]. The study was followed by the publication of a number of case studies, the majority of which precisely pointed out that people with orthorexic behavior are not mainly interested in their self-image and in maintaining their body weight, but strive for optimal health through inflexible food choices and limitations [8,9,21]. These case studies also underline that ON could be a distinct mental health condition since persons with ON might get dominated by fixative ideas over food and become socially isolated due to these obsessions [22]. Therefore, even though the concept of orthorexia is related to healthy eating, ON may become paradoxically unhealthy [15]; as opposed to “healthy orthorexia”, that pertains to a non-pathological interest in healthy eating, ON could be associated with other mental health conditions and personality traits [23].

### 3.3. ON and the Spectrum of EDs

The term “Eating Disorder (ED)” has been used to describe the presence of a persistent condition related to food intake or an eating-related behavior resulting from abnormal dietary patterns [8]. EDs may lead to significant alterations in either food consumption or nutrients’ absorption and affect one’s physical and/or mental health to a great extent, as extreme eating patterns and behaviors can afflict an individual’s life in a negative way encompassing life endangering dietary restrictions [24]. EDs’ manifestations include a range of atypical or subclinical signs, typical symptoms, personality traits, and specific attitudinal patterns [25]. Common disordered eating behaviors among adolescents and young adults include unhealthy body weight control methods through restricting diets, excessive exercise and use of dehydration techniques, vomiting, diuretics, laxatives, or diet medications, or polyphagia/hyperphagia, a term used for the constant search for food and the excessive intake of food leading to obesity, binge eating disorder, bulimia nervosa. The “EDs spectrum approach” was introduced so as to examine specific patient phenotypes, as well as the impact on everyday lives and social interactions [25].

Thus, this approach may represent a very useful tool to overcome the actual limitations of the eating disorder diagnoses [25].

The pathophysiology of EDs has undergone changes over time under the influence of various environmental and individual factors, with many new clinical entities constantly emerging, claiming a place in the spectrum of eating and behavioral disorders [25]. The fifth edition of the “Diagnostic and Statistical Manual of Mental Disorders–DSM-5” classifies all EDs in a separate chapter, in which the majority of the clinical phenotypes, such as Anorexia Nervosa (AN), Bulimia Nervosa (BN), Binge Eating Disorder (BED), Other Specified Feeding and Eating Disorders (OSFED), pica, rumination disorder, Avoidant/Restrictive Food Intake Disorder (ARFID), and other Eating Disorders Not Otherwise Specified (EDNOS) are included into the main diagnostic classes. The last category applies to conditions, in which symptoms that characterize a feeding or ED and can potentially result in clinically significant impairment of social life or other crucial fields of functioning, predominate but do not meet the full criteria [26]. ON is not currently recognized as an ED in DSM-5, since the great heterogeneity in its clinical presentations is insufficient to make a more specific diagnosis [21]. A history of EDs has shown positive correlations with ON in various studies [8,27]; accordingly, a recent systematic review and meta-analysis of 36 studies showed a significant, moderate association between ON and symptoms of EDs (pooled effect size = 0.36, 95% CI: 0.30–0.43) [28].

In recent years, there have been cases of misconceptions between the terms AN and ON and a substantial fluidity among their diagnoses has been observed [21]. Although the two conditions share similar abnormal eating attitudes, individuals suffering from AN are mostly preoccupied with body image, restriction of calories and loss of weight [14,20,21]. The eating pattern of these individuals is the expression of an inner need to prevent themselves from gaining weight and their feelings of self-confidence and satisfaction mainly rely on the image of a slim body [14]. In contrast with AN, in which the patients’ level of self-esteem is highly dependent on the amount of food they consume and the weight they manage to lose, ON is not associated with a weight-loss strategy [10]. The main difference between AN and ON lies in the motivation and the final goal: weight loss in AN versus healthy eating in ON [21]. In the latter, a lack of body image distortion is also observed and the fear of becoming fat is not necessarily related to the condition [21]. Healthy eating becomes the obsession that overshadows almost every aspect of life, as orthorexic individuals become pathologically dominated by food-centered thoughts and emphasize “healthism” as a personal responsibility in order to maintain their health through food choices [29]. “Healthism” entails a health consciousness that motivates individuals to thrive for best possible eating and exercise behavior [30]. However, orthorexic attitude could be associated with weight loss, as a result of extreme selection of certain food categories and the strict exclusion of others [31].

In addition, confusion between ON and ARFID has also been observed [9]. Although the latter is characterized by the need to follow an obsessively rigid diet designed to obtain optimal health, it is also based on the lack of interest in nutrition in general, anxiety for the appearance of nausea or other aversive consequences after consuming specific types of food, avoidance of certain foods with specific sensory characteristics, such as distinctive odor or consistency, and limited variety in dietary choices resulting in severe nutritional deficiencies [26]. Its main difference from ON is that orthorexic individuals tend to demonstrate excessive interest in meticulously excluding certain food types from their lives [25].

### 3.4. Diagnosis of ON-Questionnaires

The assessment of ON is usually feasible with the use of validated questionnaires. The very first tool introduced by Bratman and Knight to diagnose ON was Bratman’s Orthorexia Test (BOT), a 10-step rating scale which in the following years was replaced by an altered form of the scale, the ORTO-15 [32]. Numerous other tools are available, including the Eating Habits Questionnaire (EHQ), the Dusseldorf Orthorexia Scale (DOS) [33], the Barcelona Orthorexia Scale (BOS), and the Teruel Orthorexia Scale (TOS). ORTO-11 has also been used to measure the prevalence of ON [21].

Donini et al. asked their participants to fill out a 15-step questionnaire about their food-related beliefs, the potential outcomes of eating “clean” foods, their attitudes towards the selection and preparation of their meals, and the total impact these decisions have on their daily lives and social interactions. According to their scores, the participants were classified in two categories, ON or non-ON, with lower scores representing greater ON symptomatology [13]. The revised version of ORTO-15, namely ORTO-R, is a valid tool for the detection of ON [34].

Various diagnostic criteria have also been proposed for ON, converging to the pathological preoccupation with healthy nutrition, associations with mental health conditions, such as anxiety and distress, as well as changes in social life, malnutrition, and weight loss [9,16].

### 3.5. Epidemiology, Prevalence, and Sociodemographic Correlates of ON

As mentioned earlier, ON may begin innocently in many cases, associated with one’s desire to manage or prevent a disease. An ON prevalence of 15.5% in men and 11.1% in women suffering from diabetes mellitus was revealed in a Turkish study [35], while according to a study conducted in the USA by Oberle et al., higher prevalence of ON was reported among obese persons, in their attempt to lose weight and acquire a healthy body image [36]. According to the latter, except for the greater Βody Μass Ιndex (BMI), gender has also been often associated to ON.

However, the findings of numerous studies are contradictory as to whether gender differences affect the occurrence of ON or not. In contrast to AN, in which the prevalence among women is significantly higher than the one among men, with an average ratio of 9:1 [37], the results of existing research on ON are controversial [8], with some studies pointing out the greater prevalence of ON symptomatology among women [38], others reporting that men with ON outnumber women [39], whereas there are also studies finding no differences in the frequency of ON between males and females [32]. A systematic review and meta-analytical integration of 67 studies did not reveal differences in orthorexic behaviors between the two genders, with the exception of pathologically healthful eating that was slightly more prominent in women [40].

Referring to income and educational level, there are indications that the prevalence of OΝ is higher in people with high income and higher educational level, as they have easier access to products of high nutritional value, which are more expensive, and to knowledge about a healthy lifestyle [41].

Another study conducted in the U.S. encompassed 384 university students and showed a prevalence of 8.0% whereas, an additional 12.4% was considered at risk of developing ON [33]. Additionally, according to the results of the survey of Tremelling et al. among 636 registered dietitian nutritionists in the USA, the average prevalence of signs and symptoms of ON ranged between 35.0–57.8% for people belonging to high-risk groups. These groups included nutritionists, students of nutrition, medicine, nursing and other healthcare-related professions, fitness and yoga instructors, gymnasts and trainers, and even dancers and young artists. In the aforementioned study, 49.5% of the sample were found to be prone to present or have already presented ON symptomatology, because they appeared to have intense food, shape, and body concerns [42]. A Greek study by Chaniotis et al. found that orthorexic behavior mainly concerned women of high educational level (16.2%) [43]. In the same vein, Dell’Osso et al. argued that ON symptoms were more common among individuals with a higher level of education, younger than 30 years old, and mainly university students. Their findings also suggested that female university students presented a higher tendency to developing ON than males with a frequency of 53.4% and 40.6%, respectively [25]. High rates have, also, been recorded in studies concerning medical doctors and medical students in Turkey (45.5% and 43.6%, respectively) [43].

With reference to sociodemographic factors, such as age, gender, family income, educational background, and their correlations with ON, significant conclusions can be drawn from the surveys conducted in 2018 by McComb and Mills [8]. According to them, OΝ was more common at younger ages than at older ones. In a sample of 16–29 year olds in Turkey, OΝ was more frequent in people under 21 years old [39]. Similarly, another survey conducted in Italy [25] also presented a higher prevalence of ON among younger students and adolescents. However, two other studies in Italy [13] and Hungary [21] produced the opposite result, with ON being more common in older ages, whereas there were also studies that reported no significant differences according to age [11,44].

Finally, in relation to the different cultural background as a factor that might contribute to the development of ON, Strahler and associates in a sample of German and Lebanese young adults, argued that Lebanese participants were more prone to ON symptomatology than the Germans (8.4% and 4.9%, respectively), highlighting the role of Western civilization body image standards having penetrated into Middle East culture in recent years through social media [45]. Other efforts have also shown cross-cultural aspects of orthorexia and the importance of cultural elements in the construct of ON, including a recent study revealing differences between Italian, Polish, and Spanish university students [46].

### 3.6. Mental Health-Related and Nutritional Correlates of ON

A number of theories have been formulated in order to detect the risk factors of ON. Research has shown that biological, biochemical, psychological, and sociological/environmental factors may underlie the development of EDs in general, and of ON [47]. While most EDs have been reported to have their roots in childhood and early adolescence, their symptoms become usually evident in adolescence and early adulthood [48]. Recent research has also shown that the presence of stressful situations during childhood may affect the development of a wide range of EDs in adolescents [49].

According to research, people with ON or those at increased risk of developing it, typically share OCD and/or autistic disorder characteristics [25]. The possible etiological correlation of ON with OCD is based on the common features between the two syndromes [19]. Having an anxious personality, the need to have complete control, the phobia of food impurity, and a potential tendency to perfectionism are some of their shared characteristics [19,50]. A systematic review and meta-analysis published in 2021 showed a significant association between ON and OCD symptoms (pooled effect size = 0.21, 95% CI: 0.15–0.27) [28].

Perfectionism has also been reported as a risk factor not only for EDs such as AN and BN, but ON as well [2]. People with ON have been described as having excessively high demands on themselves, along with a strong sense of harsh criticism towards themselves and those around them, and a strong obsession with eliminating the elements of their personality that they perceive as defects [2,8]. Cross-cultural aspects seem to modify the association between perfectionism and ON, as evidenced by a recent Polish–Italian study on university students [51]. Interestingly, from a methodological point of view, the ability to document an association between OCD and ON involves the questionnaire implemented; a multi-center, observational, controlled study showed that the revised version of ORTO-15, namely ORTO-R, overcomes difficulties of ORTO-15 in detecting the aforementioned association [34].

Adoption of a vegetarian or vegan diet is associated with ON according to some studies. In a study conducted in the U.S., university students who were vegetarian, vegan or pescatarian, or followed paleo or gluten free diets, were reported to have a greater ON risk than those who did not follow a special diet [52]. Analyses of Italian [25], Polish [53], and German samples [54,55] also revealed that those who identified as vegetarians or vegans had a greater ON risk than the ones who did not. On the other hand, several studies have argued that vegetarianism or veganism is not related to ON, indicating that these diets are not a risk factor for ON [11,56,57]. In a study conducted by Dunn et al., the opposite results were found, showing that American university students who did not follow a special diet were more prone to ON than vegans [56].

ON has been associated with being a customer from organic food stores [58]. Apart from adopting a special diet, an increased risk for ON has been related to more rigorous exercise engagement. Studies involving Italian athletes [59] and Polish [41] and Hungarian students [21] showed that the level of involvement in sports could be a predictive factor for ON symptoms. Exercise frequency has also been associated with greater ON tendency as reported in studies among university students in the United Kingdom [60] and the U.S.; in the latter, more specifically, it was found that exercising for body and health improvement and internal motivation were positively associated with ON tendency rather than external motivation [61]. A systematic review and meta-analysis of 25 studies on 10,134 participants showed a small but significant association between ON and exercise (r = 0.12, 95% CI: 0.06–0.18) with negligible differences between genders [62].

Other possible risk factors of ON may include the urge to maintain health or to avoid illness through diet, the effort to gain control from a chaotic life or the constant desire of individuals to be part of a group; in this case, a community with very specific dietary patterns and beliefs [12].

Regarding the relationship between ON and self-image, although early studies suggest that ON is not related to the desire for weight loss, later research by Bartel et al. has argued that the features of ON could be associated with the individuals’ weight dissatisfaction as well as their attempt to regulate it through dietary choices [63]. In the case of dieticians, who exhibit particularly high rates of ON, it has not been clarified whether the origins stem from the purity of food or the overestimation of the ideal weight and lean body value [42].

Important findings about ON in young people in Greece were reported by a study conducted with the participation of students from Harokopio and other Greek universities [64]. The study, which employed the ORTO-15 and the Food Frequency Questionnaire, showed that there was a significant correlation between ON and gender, with the highest ORTO-15 values being recorded in young women (59%). Another important finding was that a large percentage of young adults who developed orthorexic behavior bought low-fat and low-calorie products, visited health food stores more often, consumed organic products, read food labels, avoided ready-made meals and fast-food restaurants, and were thoroughly informed about issues related to their diet. There was also a positive correlation between orthorexic behavior and the consumption of fruit and tea, while a negative correlation was observed with meat, bacon, and cereals. Furthermore, there was no significant difference between young adults who exhibited features of orthorexic behavior and those who did not in terms of adherence to a Mediterranean Diet [64]. Interestingly, another study in dietetics students in Greece revealed an association between orthorexic behavior, increased BMI, waist circumference, and energy intake, highlighting body image as a professional stressor for the students and their need to conform to healthy nutrition ideas as role models [65].

Various mental health conditions have been associated with more severe cases of ON. ON has been associated with symptoms of depression, insomnia, lack of interest in simple everyday activities, chronic fatigue, feelings of disappointment, and dissatisfaction in life [66]. A case series on ON clinical subjects unraveled a previous diagnosis with a spectrum of mental health conditions, namely OCD, bulimia nervosa, anxiety disorders, and psychotic disorder before the onset of ON [67]. Depression has also been reported as a risk factor of ON and other EDs and metabolic problems in adolescents and adults [68]. Abnormal eating behaviors are common among adolescents and young adults with Pervasive Developmental Disorders (PPD) and Asperger syndrome who tend to be highly selective in the color, odor, temperature, and texture of the food they consume [25,69]. ON personality traits have several similarities with the attachment to routines, the repetitive behavioral patterns, emotional distancing, and social isolation observed in adolescents and young adults with autism spectrum disorders. Ranking ON among the other disorders of the autism spectrum was also proposed [19].

ON has a bidirectional association with metabolic diseases and disorders of the endocrine system, because it could present as a cause and an outcome of such conditions [70]. On one hand, severe ON might be associated with malnutrition through deficiency of nutrients caused by the exclusion of specific kinds of food from diet [14,18], resulting in thyroid and gonadal dysfunction, dysregulation of the hypothalamic–pituitary axis [71], amenorrhea and menstrual disturbance, delayed puberty, poor acquisition of peak bone mass [70], vitamin D deficiency [72], and fluctuations of the glycemic index [70]. On the other hand, patients suffering from such endocrine diseases might also become obsessed about the pursuit of healthy eating, in order to protect themselves from additional health issues, especially if they choose to try to manage these serious conditions based on their own knowledge and experience without consultation with an expert [70,73]. A recent systematic review of six observational studies concluded that patients with diabetes mellitus often present with ON and pointed to the need for evaluation of underlying mechanisms [74].

Other physical health conditions have been associated with ON. A study in Turkey recently documented that increased risk of ON is present in breast cancer patients, in view of held beliefs that unhealthy eating causes cancer; this finding prompted the need for nutritional counselling in this vulnerable group [58]. Food allergy has been associated with ON according to a study on customers from organic food stores in Italy; the finding was evident in the EHQ score, but was not replicated in the analysis on ORTO-15 scores [58].

### 3.7. ON and Social Media as an Important Risk Factor among Adolescents and Young Adults

During the last decade social media has been an integral part of adolescents’ and young adults’ daily lives, as well as the dominant means of information and communication between them [75]. Recent studies have shown that the frequent use of the internet is linked to a negative impact on mental well-being [76] and they have further highlighted the association of social media use with body dissatisfaction, depression, and EDs [77,78]. Social media create trends and promote role models shaping almost every aspect of young people’s lives, as they are grossly approved by their peers. On one hand, media and sites that promote patterns of disordered eating are widely available to more and more adolescents and young adults, who are exposed to all kinds of online content, sometimes without having the digital literacy to confront the challenges in many cases [79,80]. On the other hand, parents might not be able to prevent their children from following, through mobile devices, accounts, or internet pages that spread self-destructive behaviors, distorted facts about eating patterns and intentionally altered concepts of healthy body image and body satisfaction [80,81,82].

Regarding ON, according to McCartney, the rise in the popularity of methods of “clean” eating in social and mainstream media is considered to be one of the most important environmental risk factors [83]. Among numerous other risk factors for the occurrence of ON [2], studies have identified the use of social media—especially Instagram—the pressure exerted by the media, and living in Western cultures as potential factors associated with ON [11,84]. In this context, the research by Turner and Lefevre has shown an increased prevalence of ON among populations that show great interest in both their appearance and their diet. More specifically, 49% of Instagram users who participated in the study met the criteria for developing ON [11]. None of the other social networks has had this pattern. According to the same study, ON often coexisted, preceded, or followed AN. Furthermore, the risk of developing orthorexic symptoms was related to the duration of the time period the users were engaged in Instagram, as well as to the influence of its famous users (known as influencers) through the content they displayed to their registered followers [11].

In many cases, Instagram is used to search for information and options regarding food, recipes, and tips related to eating experiences. In recent years, there has been a shift to a healthy diet promoted by a social media movement of clean and pure food supporters who share their preference for fruit, vegetables, and whole grains, avoidance of alcohol, shopping only in organic food stores, and exclusion of processed food [85]. Food-related posts which depict balanced meals without added fats and processed sugars seem to enjoy more acceptance and approval compared to those that show more unhealthy food choices. The large amount of this kind of information might overwhelm adolescents and young adults who do not necessarily have the critical ability to draw a distinct line between healthy and disordered eating [27].

### 3.8. Management of ON

ON is a condition that requires careful management and constructive cooperation among nutritionists, physicians, and other health care providers, who have to be capable of successfully recognizing subjects with ON, becoming familiar with ON and potential severe complications. It is common for people with ON to initially approach a nutritionist or dietician seeking advice on a healthy diet and a pure way of eating and living. Consultation with a mental health professional is often necessary [86].

Several researchers have supported the Cognitive Behavioral Therapy (CBT) guided by a well-trained therapist as the most suitable and appropriate method for ON [19]. The administration of Selective Serotonin Reuptake Inhibitors (SSRIs) like sertraline, fluoxetine, and paroxetine, that are widely used for the management of depression, often in combination with CBT, could be an option, because SSRIs have been proven to be efficient in the elimination of compulsive behaviors usually observed in orthorexic people [19,22]. A global approach also involves a nutritionist specialized in EDs to establish new, healthier dietary habits by invalidating the previous erroneous eating patterns, improving patients’ relation with food and, finally, disestablishing all the restrictive views and behaviors regarding nutrition [87]. Additionally, in cases of comorbidities, such as Cushing syndrome or morbid obesity, the approach has to be individualized and meticulously planned [88].

An effective intervention involves a multidisciplinary team including dieticians, physicians and psychotherapists who can provide a combination of medication, cognitive-behavioral therapy, and psychoeducation which can be administered with close monitoring in outpatient settings [19]. Aarnio and Lindeman argued that nutrition and health education should take into consideration the emotional aspects of food beliefs and food choices, apart from nutrients and physiology, in order to put together affective approaches in regards to counseling people with ON [89]. When psychotherapy is applied, interventions should be individualized and focused not only on what patients choose to eat but also on how they shop for, how they prepare, and how they feel about the foods they consume [19].

## 4. Discussion

The scope of the present review is to present the available literature evidence regarding features of ON. During the last 15 years, a number of studies have been implemented in order to describe the manifestations, prevalence, and physical and mental health aspects of ON. As mentioned earlier, adolescents and young adults are at risk of ON. Most studies use questionnaires that can assess symptoms of ON. The main aspects of this review article are summarized in the Graphical Abstract.

ON is a relatively recent, emerging condition characterized by a strong obsession and attachment to healthy food choices that is affecting a growing number of the population in developed countries. Although the prioritization of a healthy eating model is greatly sought, the obsession with the quality and purity of the food intake in recent years has been associated with orthorexic behaviors [8]. Experts agree that ON could become a problem in cases it represents an obstacle to the functional participation of the individual in his daily and social activities [15,21].

It is important, however, to separate the criteria that define a healthy lifestyle model from the onset of ON [19]. Individuals who make well-informed healthy choices regarding their diet cannot be characterized as having ON, as long as a healthy lifestyle and food choices do not have a negative impact on their health, their social life, or become an obsession [14].

With reference to the categorization of ON, there are opposing views on whether it should be classified as an ED or included in the OCD spectrum. Although research on ON is in its infancy, most studies argue that ON should be considered as an ED which is also closely related to symptoms of obsessive–compulsive behavior, rather than an OCD [10]. In a recent study conducted by Bartel and associates, it is argued that although ON should be classified as an ED, no safe conclusions can be drawn as to whether it is a precursor to EDs, an autonomous ED, or a disorder arising from an existing condition [63]. However, not being officially recognized as an ED, it has been classified as an EDNOS [26]. In addition, individuals who have been diagnosed with OCD or are in the autism spectrum are more likely to develop ON symptoms [24,25,69].

Understanding the risk factors related to ON features might contribute to the development of a protective framework for prone adolescents and young adults towards prevention [87]. Further longitudinal research focusing primarily on adolescents is needed to shed light to an under-researched field with a view to identifying how ON symptoms evolve along with older age. Relevant research should be carried out in in youth in order to study the risk factors of ON in different subgroups of the population. Special attention should be paid to the assessment of short- and long-term associations of ON with nutritional status, including energy and nutrient deficiencies, as well as with mental health, well-being, and overall quality of life of adolescents and young adults.

Limitations of this review include the fact that it has not been conducted with the methodology of a systematic review and that the search was limited to only two literature databases. On the other hand, a thorough effort was undertaken to include the findings of all identified relevant articles on the main aspects of ON in the broader context of the health conditions that affect children, adolescents, and young adults.

## 5. Conclusions

EDs are serious conditions with complex and multifactorial etiology that affect mental and physical health, as well as quality of life. Although careful food selection is not a pathological behavior itself, extreme dietary restrictions and social life problems should ring an alarm bell for possible ON. ON is not included in the official classification of EDs in the DSM-5 and its prevalence varies from country to country, as well as among different population groups. There is a need to expand research in relation to the intensity and the extent of orthorexic behaviors, their associations with physical, mental, and psychological health, and the role of risk factors, in order to strengthen prevention and intervention strategies.

Prevention programs should be initiated at an early age. Prevention educational programs should focus on adolescents and their parents. The programs may include information on physical development during adolescence, balanced nutrition for health maintenance, healthy ways to control body weight, health effects of unhealthy weight control methods, avoidance of negative comments and pressure regarding body weight in the context of family and peer’s environment, as well as information on professionals who may advise adolescents on nutrition and ED issues.

Moreover, the regular use of screening tools specific for EDs in general and ON in particular, as a part of the regular medical monitoring in vulnerable persons, might be another useful preventive measure in order to detect those conditions at early stages. In case of ON, the treatment protocol recommended by a multidisciplinary team should be implemented.

Future research should focus more on long-term effects of ON in adolescent and young populations. Longitudinal studies on adolescents will be of great importance, especially regarding associations that might arise in adult life. Furthermore, there is a need for additional research on specific nutrient deficiencies in relation to ON. Use of nutritional intake assessment tools and clinical interviews, in combination with ED and ON screening tools, will be useful for this purpose.

## Data Availability

Not applicable.
